# Engineering of *Escherichia coli* for D-allose fermentative synthesis from D-glucose through izumoring cascade epimerization

**DOI:** 10.3389/fbioe.2022.1050808

**Published:** 2022-10-20

**Authors:** Ling-Jie Zheng, Qiang Guo, Ya-Xing Zhang, Chen-Yang Liu, Li-Hai Fan, Hui-Dong Zheng

**Affiliations:** ^1^ College of Chemical Engineering, Fujian Engineering Research Center of Advanced Manufacturing Technology for Fine Chemicals, Fuzhou University, Fuzhou, China; ^2^ Qingyuan Innovation Laboratory, Quanzhou, China

**Keywords:** metabolic engineering, fermentation, rare sugar, biomanufacturing, cell factory

## Abstract

D-Allose is a potential alternative to sucrose in the food industries and a useful additive for the healthcare products in the future. At present, the methods for large-scale production of D-allose are still under investigation, most of which are based on *in vitro* enzyme-catalyzed Izumoring epimerization. In contrast, fermentative synthesis of D-allose has never been reported, probably due to the absence of available natural microorganisms. In this work, we co-expressed D-galactose: H^+^ symporter (GalP), D-glucose isomerase (DGI), D-allulose 3-epimerase (DAE), and ribose-5-phosphate isomerase (RPI) in *Escherichia coli*, thereby constructing an *in vivo* Izumoring pathway for yielding D-allose from D-glucose. The carbon fluxes and carbon catabolite repression (CCR) were rationally regulated by knockout of FruA, PtsG, Glk, Mak, PfkA, and PfkB involved in the pathways capable of phosphorylating D-fructose, D-glucose, and fructose-6-phosphate. Moreover, the native D-allose transporter was damaged by inactivation of AlsB, thus driving the reversible Izumoring reactions towards the target product. Fermentation was performed in the M9 medium supplemented with glycerol as a carbon source and D-glucose as a substrate. The results show that the engineered *E. coli* cell factory was able to produce approximately 127.35 mg/L of D-allose after 84 h. Our achievements in the fermentative production of D-allose in this work may further promote the green manufacturing of rare sugars.

## Introduction

D-Allose is a rare hexose sugar with ultra-low energy and 80% sucrose sweetness (Iga et al., 2010; Mooradian et al., 2017). Many researches have reported that D-allose shows certain efficacy in inhibiting cancers, such as ovarian cancer, hepatocellular carcinoma, pancreatic cancer, prostate cancer, and cervical cancer (Naha et al., 2008; Yamaguchi et al., 2008; Yokohira et al., 2008). Moreover, it can also act as an anti-inflammatory agent to alleviate cisplatin-induced nephrotoxicity (Miyawaki et al., 2012), as an antioxidant to prevent oxidative damage caused by reactive oxygen species (ROS) (Sun et al., 2006; Ishihara et al., 2011; Nakamura et al., 2011), and as an immunosuppressant in cryoprotection of biological cells and tissues. These beneficial physiological properties make D-allose a potential sweetener in food and healthcare products, and the production of D-allose has been gradually becoming a research focus (Lim and Oh, 2011).

The routes for D-allose synthesis are either chemical or biological, of which chemical process normally generates a variety of by-products, and the harmless disposal of the resulting waste also remains a challenge (Wang et al., 2020; Morimoto et al., 2022; Vigo et al., 2022). In contrast, biosynthesis has the advantages of high specificity, mild conditions, and environmental friendliness, and is in line with the concept of green manufacturing. Currently, most of the studies on D-allose bioproduction are based on the Izumoring enzymatic cascade (Granström et al., 2004; Izumori, 2006). Izumoring was proposed in 2004 as a strategy to synthesize rare sugars through enzyme-catalyzed epimerization between monosaccharides (Granström et al., 2004). Although reversible epimerization leads to the low conversion of substrates, this method is still the best choice for preparation of rare sugars, and has been successfully applied in the industrial production of D-allulose, a stereoisomer of D-fructose (Zhu et al., 2012; Su et al., 2018). However, it is interesting that Izumoring synthesis of rare sugars is basically achieved through *in vitro* biocatalysis rather than cell factories (Kim et al., 2006; Zhu et al., 2012). The reason may be that the substrate for Izumoring is usually D-fructose or D-glucose, which can be efficiently phosphorylated by cells, thus being allowed to enter the central metabolic pathways as a carbon source for growth (Guggisberg et al., 2014; Luo et al., 2014). Therefore, although fermentation has more cost-reducing potential than enzymatic catalysis for D-allose production, metabolic engineering of cell factories is quite important, especially the phosphorylation pathways of substrate and intermediates should be rationally reprogrammed.

**FIGURE 1:**
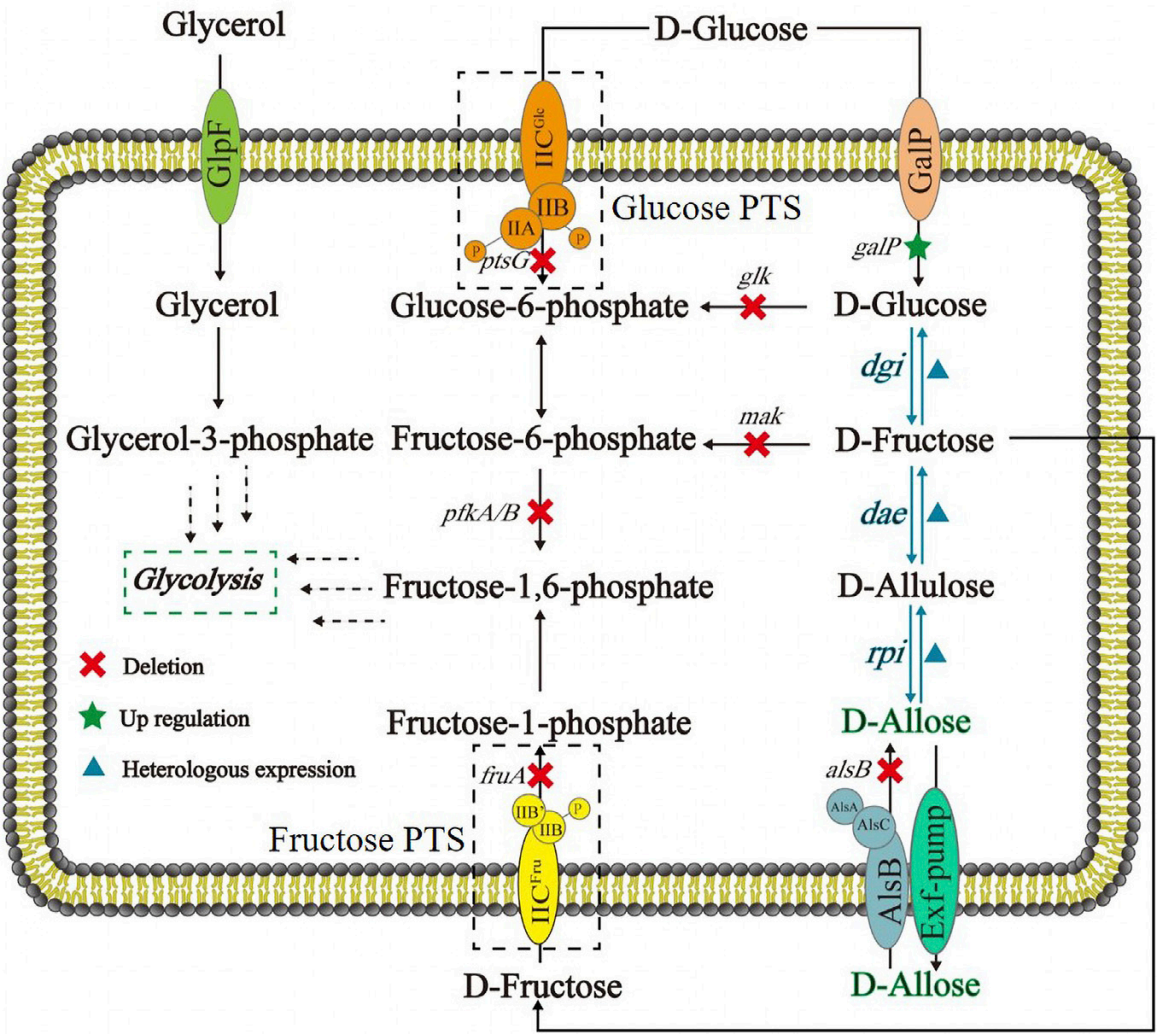
Schematic of *E. coli* cell factory for synthesizing D-allose from D-glucose based on Izumoring cascade epimerization. DGI: D-glucose isomerase; DAE: D-allulose 3-epimerase; RPI: ribose-5-phosphate isomerase; GalP: D-galactose H^+^ symporter; Glucose PTS: the phosphoenolpyruvate transferase system of D-glucose; Fructose PTS: the phosphoenolpyruvate transferase system of D-fructose.

Here we designed and constructed an *Escherichia coli* cell factory capable of synthesizing D-allose from D-glucose (Figure 1), thereby demonstrating that it is possible to produce rare sugars by use of *in vivo* Izumoring cascade epimerization (Lim and Oh, 2011; Chen et al., 2018). Briefly, to ensure that D-glucose was available in cells as a substrate in its unphosphorylated form, the phosphoenolpyruvate: carbohydrate phosphoenolpyruvate transferase system (PTS) that can transport and concomitantly phosphorylate D-glucose were replaced with GalP, a D-galactose: H^+^ symporter involved in D-glucose uptake (Luo et al., 2014). D-Glucose isomerase (DGI) (Liu et al., 2015; Jin et al., 2021), D-allulose 3-epimerase (DAE) (Kim et al., 2008), and ribose-5-phosphate isomerase (RPI) (Park et al., 2007; Yeom et al., 2011) were co-expressed to perform the reactions from D-glucose to D-allose, in which D-fructose and D-allulose were intermediates of the cascade epimerization. The kinases catalyzing the phosphorylation of D-glucose and D-fructose were knocked out, and the Embden-Meyerhof-Parnas (EMP) pathway was blocked, with the purpose to maximize the titer of the target product. In this work, our achievements provide an alternative strategy for D-allose synthesis, and may promote the research on fermentative production of rare sugars.

## Materials and methods

### Strains and media

The strains used in this study were listed in Table 1. *E. coli* JM109 (DE3) purchased from LMAI Bio (Shanghai) was the host for D-allose production. Luria–Bertani (LB) medium was composed of 10 g/L sodium chloride, 10 g/L tryptone, and 5 g/L yeast extract. The M9 medium for fermentation contained (per L): 4.78 g Na_2_HPO_4_, 2.99 g KH_2_PO_4_, 0.5 g NaCl, 0.12 g Mg_S_O_4_, 33 mg CaCl_2_, 1 μg Thiamin, 1 μg Biotin, 50 mg EDTA, 0.1 mg H_3_BO_3_, 0.13 mg CuCl_2_, 0.84 mg ZnCl_2_, 8.3 mg FeCl_3_, 0.1 mg CoCl_2_, and 16 μg MnCl_2_. D-Glucose and glycerol were added in M9 medium for D-allose synthesis and cell growth, respectively.

**TABLE 1:** Strains and plasmids used in this study.

Name	Relevant characteristics	References
Strains		
*E. coli* JM109 (DE3)	Wild type *E. coli*	LMAI Bio
*E. coli* (Empty A)	*E. coli* JM109 (DE3) harboring pETDuet-1	this study
*E. coli* (Empty B)	*E. coli* JM109 (DE3) harboring pETDuet-1 and pRSFDuet-1	this study
*E. coli* (DGI)	*E. coli* JM109 (DE3) harboring pETDuet-*dgi*	this study
*E. coli* (DAE)	*E. coli* JM109 (DE3) harboring pETDuet-*dae*	this study
*E. coli* (RPI)	*E. coli* JM109 (DE3) harboring pETDuet-*rpi*	this study
*E. coli* (DGI, DAE, RPI, GalP)	*E. coli* JM109 (DE3) harboring pETDuet*-galP*-*dgi* and pRSFDuet-*dae*-*rpi*	this study
*E. coli* (DGI, GalP)	*E. coli* JM109 (DE3) harboring pETDuet-*galP*-*dgi*	this study
*E. coli* (DGI, GalP, ΔFruA)	*E. coli* JM109 (DE3) harboring pETDuet-*galP*-*dgi* with *fruA* deleted	this study
*E. coli* (DGI, GalP, ΔFruA, ΔPtsG)	*E. coli* JM109 (DE3) harboring pETDuet-*galP*-*dgi* with *fruA* and *ptsG* deleted	this study
*E. coli* (DGI, GalP, ΔFruA, ΔPtsG, ΔGlk, ΔMak, ΔPfkA, ΔPfkB)	*E. coli* JM109 (DE3) harboring pETDuet-*galP*-*dgi* with *fruA*, *ptsG*, *glk*, *mak*, *pfkA*, and *pfkB* deleted	this study
*E. coli* (DGI, DAE, RPI, GalP, ΔFruA, ΔPtsG, ΔGlk, ΔMak, ΔPfkA, ΔPfkB)	*E. coli* JM109 (DE3) harboring pETDuet-*galP*-*dgi* and pRSFDuet-*dae*-*rpi* with *fruA*, *ptsG*, *glk*, *mak*, *pfkA*, and *pfkB* deleted	this study
*E. coli* (DGI, DAE, RPI, GalP, ΔFruA, ΔPtsG, ΔGlk, ΔMak, ΔPfkA, ΔPfkB, ΔAlsB)	*E. coli* JM109 (DE3) harboring pETDuet-*galP*-*dgi* and pRSFDuet-*dae*-*rpi* with *fruA*, *ptsG*, *glk*, *mak*, *pfkA*, *pfkB*, and *alsB* deleted	this study
Plasmids		
pETDuet-1	T7 promoter, ampicillin resistance	Novagen
pRSFDuet-1	T7 promoter, kanamycin resistance	Novagen
pETDuet-*dgi*	pETDuet-1 expressing *dgi*	this study
pETDuet-*dae*	pETDuet-1 expressing *dae*	this study
pETDuet-*rpi*	pETDuet-1 expressing *rpi*	this study
pETDuet-*galP*-*dgi*	pETDuet-1 co-expressing *galP* and *dgi*	this study
pRSFDuet-*dae*-*rpi*	pRSFDuet-1 co-expressing *dae* and *rpi*	this study
pKD46, pCP20, pKD13	λ red recombination system	Guo et al. (2021)

### Plasmid construction

The plasmids of pRSFDuet-1 and pETDuet-1 for co-expressing protein were purchased from Novagen (Table 1). The genes of *dgi* (GenBank: AAA26838.1) (Jin et al., 2021), *dae* (GenBank: AAK88700.1) (Li et al., 2020), and *rpi* (GenBank: ABN53797.1) (Yeom et al., 2011) were optimized and synthesized by Beijing Genomics Institute (BGI, Beijing), which were then amplified by use of PrimeSTAR DNA polymerase (Takara). The genes of *dgi*, *dae*, *rpi*, and *galP* were cloned using primers *dgi*-F and *dgi*-R, *dae*-F and *dae*-R, *rpi*-F and *rpi*-R, *galP*-F and *galP*-R (Table 2), respectively, then digested with *Nde* I and *Xho* I, *Mfe* I and *kpn* I, *BamH* I and *Hind* III, *Nco* I, and *Hind* III (NEB, Beijing) at 37°C for 1 h. The obtained DNA fragments were ligated to pETDuet-1 or pRSFDuet-1 with the help of T4 DNA ligase (NEB, Beijing) at 16°C for 12 h, resulting in pETDuet-*dgi*, pRSFDuet-*dae*, pRSFDuet-*rpi*, pETDuet-*galP*-*dgi*, and pRSFDuet-*dae*-*rpi*, respectively.

**TABLE 2:** Primers applied in gene amplification and knockout.

Name	Primer sequence (5′-3′)	Gene
Gene amplification		
*galP*-F	CAT​GCC​ATG​GCG​ATG​CCT​GAC​GCT​AAA​AAA​CAG​GGG	*galP*
*galP*-R	CCA​AGC​TTC​CTT​AAT​CGT​GAG​CGC​CTA​TTT​CGC​GC	
*dgi*-F	GGC​ATA​TGA​ACT​ACC​AGC​CGA​CCC​C	*dgi*
*dgi*-R	CTC​GAG​TTA​GCC​ACG​GGC​ACC​CAG​C	
*dae*-F	CAA​TTG​CAT​GAA​GCA​CGG​CAT​TTA​TTA​CAG​C	dae
*dae*-R	GGG​GTA​CCT​TAG​CCG​CCC​AGA​ACA​AAA​CGG	
*rpi*-F	CGG​GAT​CCG​ATG​AAA​ATT​GGC​ATT​GGC​AGC​GAT​C	*rpi*
*rpi*-R	CGG​AAG​CTT​TCA​CTT​GCT​GTA​CTT​TTT​TTC​AAT​TTC​GCC	
Gene deletion		
*fruA*-F	GGC​ATA​ATG​AAA​ACG​CTG​CTG​ATT​ATT​GAC​GCT​AAT​CTC​GGT​CAG​GCA​CGA​TTC​CGG​GGA​TCC​GTC​GAC​C	*fruA*
*fruA*-R	ATT​ACG​CTG​CTT​TCG​CTA​CTG​CGT​CCA​CTT​CCG​GAC​GTT​TCA​GGA​AGG​CAA​GCG​ATT​GTG​TAG​GCT​GGA​GCT​GC	
*ptsG*-F	CCC​ATA​CTC​AGG​AGC​ACT​CTC​AAT​TAT​GTT​TAA​GAA​TGC​ATT​TGC​TAA​CCA​TTC​CGG​GGA​TCC​GTC​GAC​C	*ptsG*
*ptsG*-R	AGT​CTC​CCC​AAC​GTC​TTA​CGG​ATT​AGT​GGT​TAC​GGA​TGT​ACT​CAT​CCA​TCA​GCG​ATT​GTG​TAG​GCT​GGA​GCT​GC	
*pfkA*-F	GTT​CAG​AGG​TAG​TCA​TGA​TTA​AGA​AAA​TCG​GTG​TGT​TGA​CAA​GCG​GCG​GTA​TTC​CGG​GGA​TCC​GTC​GAC​C	*pfkA*
*pfkA*-R	CGA​AAT​CAT​TAA​TAC​AGT​TTT​TTC​GCG​CAG​TCC​AGC​CAG​TCA​CCT​TTG​AAA​GCG​ATT​GTG​TAG​GCT​GGA​GCT​GC	
*pfkB*-F	CTG​ATT​CGG​TGC​CAG​ACT​GAA​ATC​AGC​CTA​TAG​GAG​GAA​ATG​ATG​GTA​CGT​ATC​ATT​CCG​GGG​ATC​CGT​CGA​CC	*pfkB*
*pfkB*-R	GTT​GGT​GAT​GAT​TCC​CCC​AAT​GCT​GGG​GGA​ATG​TTT​TTG​TTA​GCG​GGA​AAG​GAG​CGA​TTG​TGT​AGG​CTG​GAG​CTG​C	
*glk*-F	CTT​TAG​CGG​AGC​AGT​TGA​AGA​ATG​ACA​AAG​TAT​GCA​TTA​GTC​GGT​GAT​GTG​GGC​ATT​CCG​GGG​ATC​CGT​CGA​CC	*glk*
*glk*-R	CCC​GAT​ATA​AAA​GGA​AGG​ATT​TAC​AGA​ATG​TGA​CCT​AAG​GTC​TGG​CGT​AAA​TGT​GCA​GCG​ATT​GTG​TAG​GCT​GGA​GCT​GC	
*mak*-F	CTA​CGC​TAT​TGA​TAT​TGA​AAA​AAA​TAA​GGA​GAG​TAC​CGT​GCG​TAT​AGG​TAT​CAT​TCC​GGG​GAT​CCG​TCG​ACC	*mak*
*mak*-R	CAT​GAT​GCG​CCA​ATT​GCC​TAC​GTT​TTT​TAC​TCT​TGT​GGC​CAT​AAC​CAC​GCA​GCG​ATT​GTG​TAG​GCT​GGA​GCT​GC	
*alsB*-F	GCA​TCA​TCA​TCC​GGC​ATC​ATT​CAG​TTT​TAT​TGA​GTG​ACC​AGG​ATT​GAA​TCA​GCG​ATT​GTG​TAG​GCT​GGA​GCT​GC	*alsB*
*alsB*-R	CTC​GGC​AAG​AAT​ATT​ACA​ACT​AAC​TTT​GCT​GGA​CGC​TTT​TTT​TGT​CTC​TGA​TTC​CGG​GGA​TCC​GTC​GAC​C	

### Gene knockout

The genes of *fruA*, *ptsG*, *glk*, *mak*, *pfkA*, and *pfkB* located on the *E. coli* genome were knocked out by a λ red homologous recombination system (Datsenko and Wanner, 2000). The kan^R^ gene with two FRT sites were amplified by use of pKD13 as a template, and then electrotransferred to *E. coli* strains harboring pKD46. After the replacement of the target gene by the kan^R^ gene with the FRT sites, plasmid pCP20 was employed to express the DNA recombinase (FLP) for eliminating the kan^R^ gene (Baba et al., 2006; Dugar et al., 2016; Yang et al., 2018). The primers for gene knockout were shown in Table 2.

### Enzyme analysis

*E. coli* strains were cultured in 100 ml LB medium with ampicillin (100 μg/ml) at 37°C and 220 rpm for 4 h. Protein expression was induced by the addition of 0.2 mM isopropyl-β-D-thiogalactoside (IPTG) when the cell density (OD_600_) reached ≈0.6. DGI, DAE, and RPI were expressed at 37°C and 220 rpm for 12 h, respectively. Then, *E. coli* strains were centrifuged at 8,000 *g* and 4°C for 10 min, which were washed with Tris-HCl (50 mM, pH 7.0) for two times, and finally suspended with 15 ml Tris-HCl (50 mM, pH 7.0). The cells were broken by ultrasonic crushing instrument JY92-IIN Jingxin co., ltd. (Shanghai) with ice-water bath for 5 min (3 s on and 3 s off). The cell debris was removed by centrifugation at 8,000 *g* and 4°C for 10 min. Protein in the supernatant was analyzed by use of 12% PAGE protein prefabricated gel (KeyGEN Biotech, Nanjing). Proteins were analyzed by MicroSpectrophotometer K5500PLus from Kaiao Technology co. ltd. (Beijing). The activity analysis was carried out at 30°C in a reaction system (500 μl) containing 400 μl Tris-HCl buffer (50 mM, pH 7.0), proper sugar (10.8 g/L D-glucose, 10.1 g/L D-fructose, or 7.8 g/L D-allulose), and 100 μL crude enzyme. After the system was preheated at 30°C for 5 min, crude enzyme was added. The reaction was terminated by boiling at 2, 5, 8, 10, 15, 30, 60, 90, and 180 min, respectively. The supernatant was obtained by centrifugation at 6,000 *g* for 10 min, and then detected by high performance liquid chromatography (HPLC). One unit of DGI, DAE, or RPI activity (U/mg) was defined as the amount of producing 1 μmol of D-fructose, D-allulose or D-allose per minute, respectively.

### Fermentation

*E coli* strains were incubated in 4 ml LB medium overnight at 37°C and 220 rpm, then cultured in 50 ml M9 medium containing appropriate D-glucose and glycerol with 100 μg/ml ampicillin and 50 μg/ml kanamycin. After the cell density (OD_600_) reached 0.6, IPTG (0.2 mM) was added for induction, and the fermentation temperature was adjusted to 30°C. Fermentation samples were taken at an interval of 12 h. The cell density was measured with ultraviolet spectrophotometer. D-Glucose, glycerol, D-fructose, D-allulose, and D-allose were analyzed by a high-performance liquid chromatograph (HPLC, HITACHI) with a refractive index detector monitor. A column of Sugar-Pak™ I purchased from Waters was employed with a mobile phase (deionized water) flow rate of 0.5 ml/min at 85°C. The injection volume of the sample was 10 μl, with a retention time of 20 min. The peaks of D-glucose, glycerol, D-fructose, D-allulose and D-allose appeared at 9.7, 13.1, 11.3, 14.9, and 12.6 min, respectively. The target product D-allose was further verified by use of liquid chromatography-mass spectrometry (LC-MS) with Agilent 6,520 and Agilent 1,260 instruments. The conditions of LC were the same as HITACHI. The conditions of MS were as follows: mode, ESI (-); scan range, 100–310 m/z; capillary voltage, 3.5 kV; fragmentor voltage, 140 V; atomization pressure, 40 psi; gas, N_2_; and gas temperature, 350°C. The standard substance of D-allose (purity ≥97%) was purchased from Yuanye Biotechnology (Shanghai).

## Results and discussion

### Rational design of an izumoring pathway for converting D-glucose to D-allose

As shown in Figure 1, the route designed for *in vivo* generation of D-allose was based on a three-step cascade of Izumoring reactions by use of D-glucose as a substrate. D-Glucose isomerase (DGI) was employed to convert D-glucose to D-fructose. This enzyme is also called D-xylose isomerase, and has been widely applied in industrial production of High-Fructose Corn Syrup (HFCS) (Liu et al., 2015), especially the DGI of *Streptomyces rubiginosus*. Although *S. rubiginosus* DGI exhibits good thermal stability, its activity is fully activated under alkaline conditions and rapidly decreases below pH 7.5, which is not conducive to its application in D-glucose isomerization in *E. coli* cells. Therefore, a *S. rubiginosus* DGI mutant with D56N and E221A was used in this work, whose catalytic efficiency has been reported to be significantly improved around neutral pH (Jin et al., 2021). The reaction from D-fructose to D-allulose can be carried out by D-tagatose 3-epimerase (DTE) or DAE. Compared with DTE, DAE has higher substrate specificity but lower thermal stability. Despite that, DAE is theoretically amenable to mild fermentation conditions, so we selected a DAE from *Agrobacterium tumefaciens*, which has been shown to be well expressed in *E. coli* (Kim et al., 2006). The synthesis of D-allose from D-allulose can be accomplished by L-rhamnose isomerase (LRhI), galactose-6-phosphate isomerase (GPI) or RPI, of which LRhI and GPI normally catalyze the formation of D-altrose (> 8%) as a byproduct (Yeom et al., 2011). Therefore, here we used a *Clostridium thermocellum* RPI with a R132E mutation, which has been reported to have a higher specific activity and catalytic efficiency for D-allulose than the wild-type enzyme (Lim and Oh, 2011; Yeom et al., 2011).

### Expression and functionality of the enzymes involved in cascade epimerization

**FIGURE 2:**
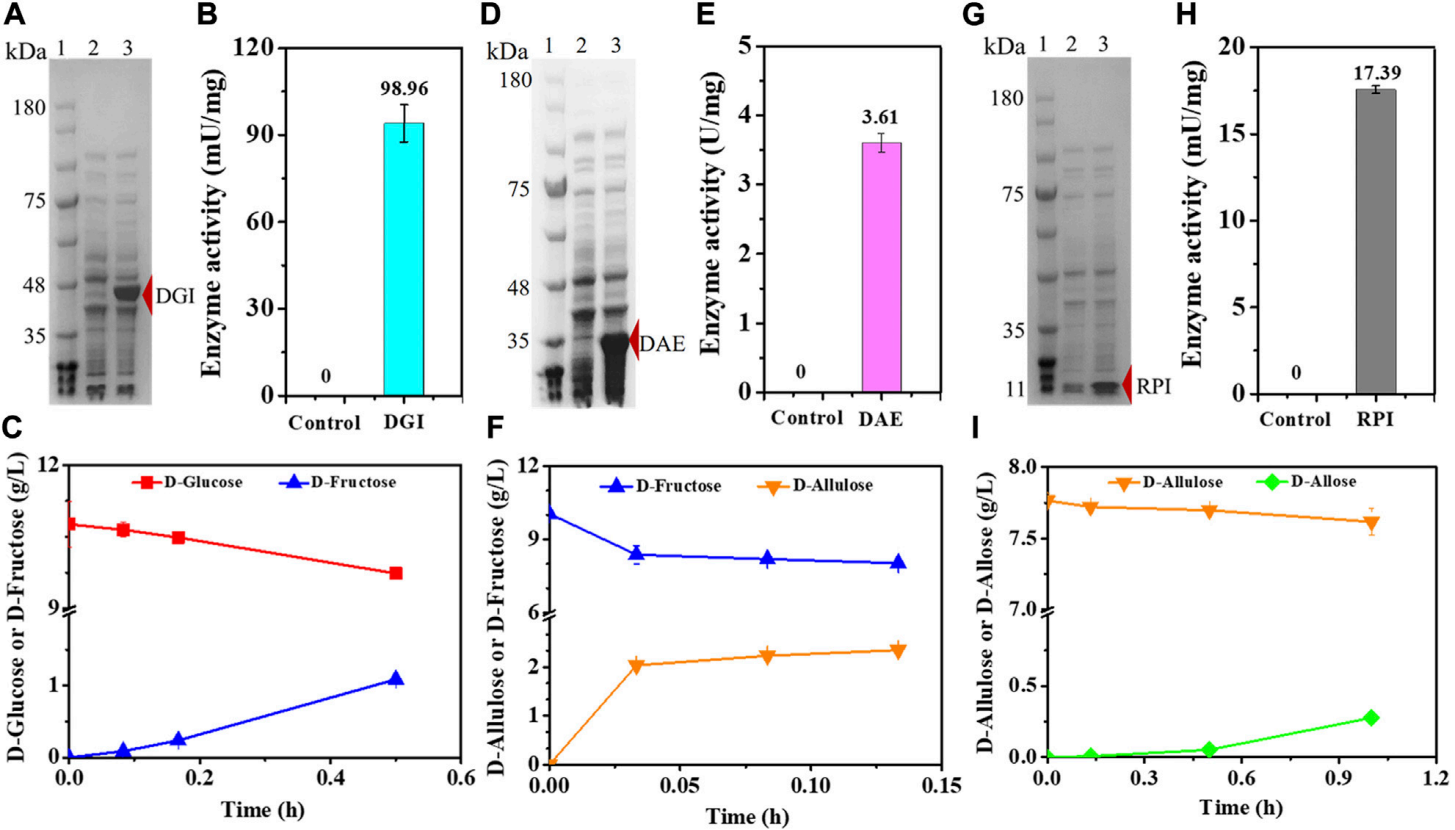
Expression and functionality of DGI, DAE, and RPI. **(A)** SDS-PAGE analysis of DGI. Lane 1: Marker, Lane 2: *E. coli* (Empty A), Lane 3: *E. coli* (DGI). **(B)** Activity of crude DGI. The supernatant of disrupted *E. coli* (Empty A) was used as a control. **(C)** Conversion of D-glucose to D-fructose by crude DGI. **(D)** SDS-PAGE analysis of DAE. Lane 1: Marker, Lane 2: *E. coli* (Empty B), Lane 3: *E. coli* (DAE). **(E)** Activity of crude DAE. The supernatant of disrupted *E. coli* (Empty B) was used as a control. **(F)** Conversion of D-fructose to D-allulose by crude DAE. **(G)** SDS-PAGE analysis of RPI. Lane 1: Marker, Lane 2: *E. coli* (Empty B), Lane 3: *E. coli* (RPI). **(H)** Activity of crude RPI. The supernatant of disrupted *E. coli* (Empty B) was used as a control. **(I)** Conversion of D-allulose to D-allose by crude RPI. Error bars indicated standard error (*n* = 3).

In order to confirm that the cascade epimerization from D-glucose to D-allose could be realized *in vivo*, we heterologously expressed DGI, DAE, and RPI in *E. coli*, respectively, resulting in the strains *E. coli* (DGI), *E. coli* (DAE), and *E. coli* (RPI). Genes were expressed under T7 promoter with IPTG as an inducer at 37°C. Then, the catalytic function of each enzyme was tested. As shown in Figures 2A,D,G, the clear bands in SDS-PAGE indicate that DGI, DAE, and RPI could be produced in cells in soluble forms with molecular masses of around 45, 35, and 15 kDa, respectively, which are in good agreement with the values (43, 32, and 17 KDa) deduced from the amino acid sequences. When D-glucose (Figure 2B), D-fructose (Figure 2E) or D-allulose (Figure 2H) was used as a substrate, the crude DGI, DAE, and RPI were able to yield D-fructose, D-allulose, and D-allose in Tris-HCl buffer (50 mM, pH 7.4) at 30°C, with activities of 98.96 mU/mg, 3.61 U/mg, and 17.39 mU/mg, respectively. These data suggest that the designed route for cascade epimerization of D-glucose to D-allose by *E. coli* was basically feasible under fermentation conditions, and the conversion of D-allulose to D-allose should be the rate-limiting step since the expression level and the activity of RPI were both lower than those of DGI and DAE.

**FIGURE 3:**
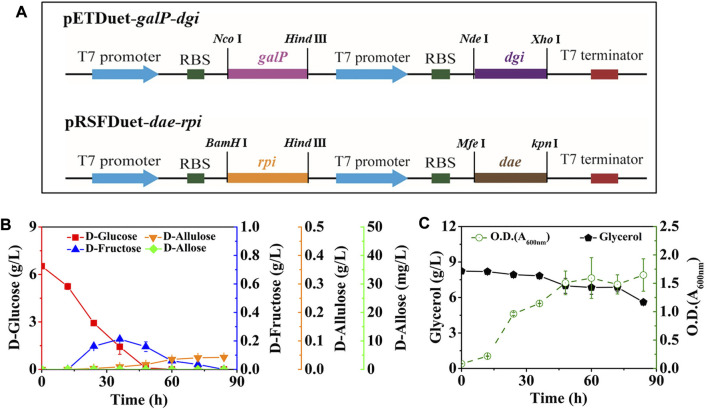
Synthesis of D-allose by fermentation using *E. coli* (DGI, DAE, RPI, GalP). *E. coli* (DGI, DAE, RPI, GalP) was cultured in M9 medium with D-glucose and glycerol at 30°C. **(A)** Recombinant plasmids of pETDuet-*galP*-*dgi* and pRSFDuet-*dae*-*rpi*. **(B)** D-Glucose, D-fructose, D-allulose, and D-allose. **(C)** Glycerol and cell density. Error bars indicated standard error (*n* = 3).

After that, we co-expressed DGI, DAE, RPI, and GalP, resulting in the strain *E. coli* (DGI, DAE, RPI, GalP). GalP is known as a D-galactose: H^+^ symporter, which is also able to transport D-glucose in *E. coli*. This passage is not accompanied by phosphorylation (Luo et al., 2014), so that the D-glucose taken up by GalP can serve as a precursor for D-fructose synthesis. The mutant was cultivated in M9 medium containing 6.52 g/L D-glucose and 8.23 g/L glycerol, with the aim of allowing cells to utilize D-glucose as a substrate for D-allose synthesis as much as possible, rather than a growth carbon source, thereby improving the yield of the product. The intermediates of D-fructose and D-allulose began to appear in the medium after 12 h of fermentation (Figure 3B). The D-fructose level first increased and then decreased, reaching a peak of 0.21 g/L at 36 h, while D-allulose gradually increased with time, reaching 0.04 g/L after 84 h. Unfortunately, we did not find the generation of the target product D-allose, and the utilization of glycerol might be limited by carbon catabolite repression (CCR), since glycerol level remained unless D-glucose was depleted (Figure 3C).

### Blocking of the phosphorylation acting on D-glucose and key intermediates

Because the activities of DGI, DAE, and RPI under fermentation conditions have been confirmed, we suspected that sugar phosphorylation might be the main reason for the failure of D-allose production, which has also been reported to be responsible for causing CCR (Fox and Prather, 2020). As an important intermediate, whether D-fructose could be continuously generated in cells was the key to synthesizing D-allose. We thus utilized the strain *E. coli* (DGI), which had a pathway capable of yielding D-fructose from D-glucose, to optimize the pathways related to the sugar phosphorylation in order to enhance D-fructose generation.

**FIGURE 4:**
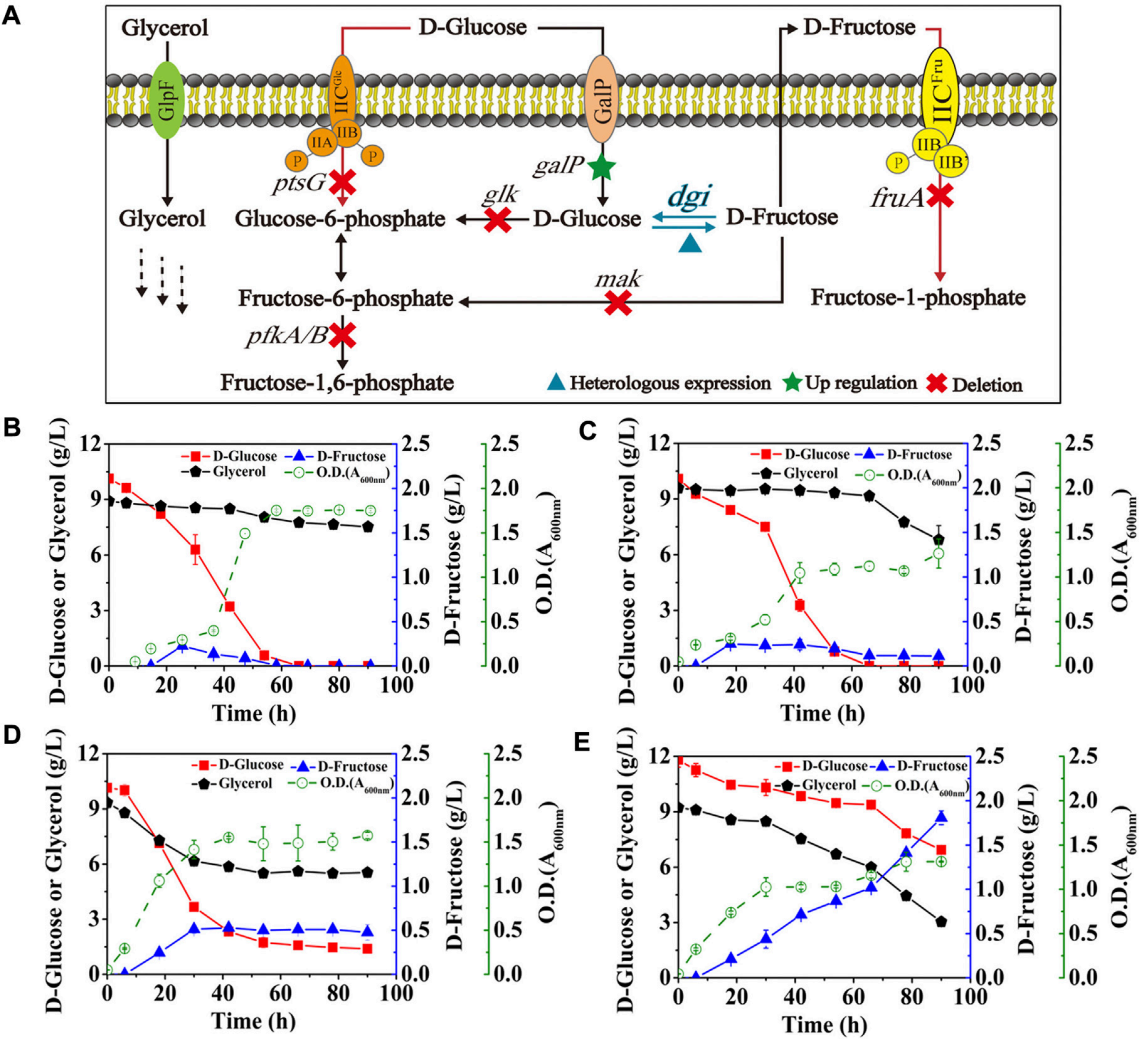
Effects of the phosphorylation acting on D-glucose and key intermediates. *E. coli* cells were cultured in M9 medium with D-glucose and glycerol at 30°C. **(A)** Schematic of the cell factory for D-fructose generation. **(B)** *E. coli* (DGI, GalP), **(C)** *E. coli* (DGI, GalP, ΔFruA), **(D)** *E. coli* (DGI, GalP, ΔFruA, ΔPtsG), **(E)** *E. coli* (DGI, GalP, ΔFruA, ΔPtsG, ΔGlk, ΔMak, ΔPfkA, and ΔPfkB). Error bars indicated standard error (*n* = 3).

*E. coli* has been reported to take up D-glucose primarily through the glucose phosphoenolpyruvate transferase system (PTS) (Phue et al., 2005), which transports and concomitantly phosphorylates D-glucose to glucose-6-phosphate, thus allowing D-glucose to enter the EMP pathway as a carbon source for growth. However, expression of GalP in *E. coli* (DGI) did not obviously improve D-fructose production and glycerol co-utilization (Figure 4B) when compared with the data in Figure 3. Fructose PTS is the major route for uptake of D-fructose by *E. coli*. It is able to phosphorylate D-fructose to fructose-1-phosphate, followed by entering the EMP pathway as a carbon source, which can explain that the produced D-fructose would be gradually consumed in the middle and late stages of fermentation (Figure 4B). Also, CCR still occurs in theory due to the presence of fructose PTS, thus inhibiting the use of glycerol by cells. Therefore, we then deleted the gene of *fruA* involved in the fructose PTS, resulting in the strain *E. coli* (DGI, GalP, ΔFruA). As illustrated in Figure 4C, inactivation of fructose PTS effectively reduced the consumption of D-fructose, which could be maintained at 0.11 g/L even after 90 h. Meanwhile, the consumption of glycerol by *E. coli* (DGI, GalP, ΔFruA) was increased when compared with that of *E. coli* (DGI, GalP), which might be because the extracellular D-fructose could not be utilized without FruA, forcing the cells to use glycerol as a carbon source. We observed that the ability of *E. coli* (DGI, GalP, ΔFruA) to metabolize glycerol was severely inhibited by D-glucose, suggesting that the expression of GalP was not able to relieve glucose CCR. Therefore, the gene of *ptsG* involved in the glucose PTS was further deleted, resulting in the strain *E. coli* (DGI, GalP, ΔFruA, ΔPtsG). The data in Figure 4D show that D-glucose consumption for growth was reduced and glycerol utilization was further increased after the glucose PTS was inactivated. More importantly, D-fructose level was increased nearly 4-fold to approximately 0.48 g/L. However, the yield of D-fructose on D-glucose was still quite low, which was less than 0.06 g/g. We then further knocked out the genes of *glk*, *mak*, *pfkA*, and *pfkB* to block the phosphorylation of D-glucose and D-fructose in the cytoplasm and the EMP pathway (Figure 1). As a result, the obtained strain had a significant improvement in D-fructose synthesis, with a D-fructose level of 1.81 g/L and a yield of 0.37 g/g on D-glucose (Figure 4E).

### Regulation of D-allose transport to enhance the forward epimerization reactions

**FIGURE 5:**
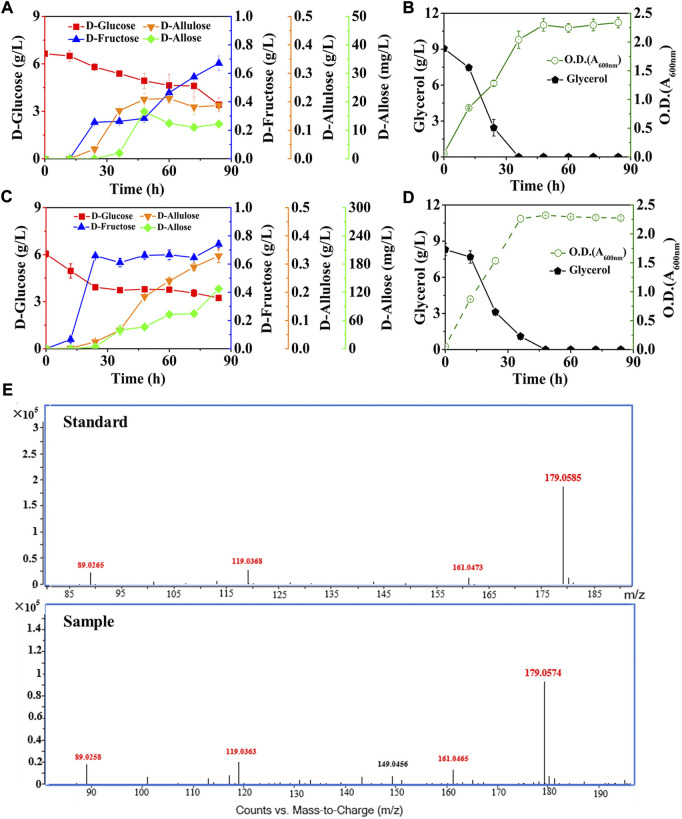
Deletion of D-allose transporter to improve the performance of cell factory. *E. coli* cells were cultured in M9 medium with D-glucose and glycerol at 30°C. **(A)** and **(B)** *E. coli* (DGI, DAE, RPI, GalP, ΔFruA, ΔPtsG, ΔGlk, ΔMak, ΔPfkA, and ΔPfkB). **(C)** and **(D)** *E. coli* (DGI, DAE, RPI, GalP, ΔFruA, ΔPtsG, ΔGlk, ΔMak, ΔPfkA, ΔPfkB, and ΔAlsB). **(E)** LC-MS analysis of D-allose standard and the fermentation sample by using *E. coli* (DGI, DAE, RPI, GalP, ΔFruA, ΔPtsG, ΔGlk, ΔMak, ΔPfkA, ΔPfkB, and ΔAlsB) at 60 h. Error bars indicated standard error (*n* = 3).

Next, we co-expressed DAE and RPI in the optimized strain, resulting *E. coli* (DGI, DAE, RPI, GalP, ΔFruA, ΔPtsG, ΔGlk, ΔMak, ΔPfkA, and ΔPfkB), and cultivated the mutant in the M9 medium supplemented with around 6.00 g/L D-glucose and 9.00 g/L glycerol (Figures 5A,B). The data show that D-allose started to appear after 24 h of fermentation, and reached its maximal level of 16.57 mg/L at 48 h, then decreased to around 12.19 mg/L. Similarly, the intermediate D-allulose also had a slight decrease in the late stage of fermentation, finally reaching 0.19 g/L at 84 h. At the same time, the D-fructose produced was 0.67 g/L, while the consumption of D-glucose was about 3.21 g/L. These data indicate that the total level of the target and intermediate products was less than 0.99 g/L when fermentation was completed, which was much lower than the amount of the substrate consumed. There are no differences in the molecular masses of D-glucose, D-fructose, D-allulose and D-allose. Thus, it suggests that most of the consumed D-glucose flowed to other pathways rather than the designed pathway for yielding D-allose, probably due to the existence of unknown pathways in *E. coli* that could bypass the genes we deleted to utilize the hexoses involved in D-allose synthesis.

Here, the three reactions involved in the Izumoring cascade epimerization were all reversible, so one of the ways capable of further improving D-allose production was to make the effluxed D-allose unable to enter the mutant cells, thereby continuously driving the reversible reactions to the forward direction. It has been reported that the D-allose transport system of *E. coli* is composed of AlsA, AlsB, and AlsC (Kim et al., 1997; Poulsen et al., 1999), wherein AlsB is responsible for the specific binding of D-allose, followed by the transmembrane passage with the aid of AlsA and AlsC. We thus knocked out the gene of *alsB* to damage the D-allose transport system of *E. coli* (DGI, DAE, RPI, GalP, ΔFruA, ΔPtsG, ΔGlk, ΔMak, ΔPfkA, and ΔPfkB), and cultivated the mutant in M9 medium under similar conditions to our previous experiments (Figures 5C,D). It is observed that the D-allose produced, which was further confirmed by high-performance liquid chromatography-mass spectrometry (LC-MS) (Figure 5E), was 10-fold higher than that of the strain without AlsB deletion (Figure 5A), reaching 127.35 mg/L after 84 h, with a yield of over 0.045 g/g on D-glucose. More excitingly, the level of D-allose rose dramatically in the middle and late stages of fermentation, and maintained an obvious upward trend even at the end of the experiment. To the best of our knowledge, this work is the first report of D-allose production from D-glucose by microbial fermentation. However, it is similar to enzymatic methods that the D-allose yield through fermentation was quite low. In this work, three reversible isomerization reactions were involved in the pathway synthesizing D-allose, which might seriously affect the yield of D-allose on D-glucose. In the future, we plan to introduce the phosphorylation-dephosphorylation pathway to convert D-glucose into D-allulose (Li et al., 2021), and then use RPI to isomerize D-allulose into D-allose, so as to further optimize the biosynthetic pathway.

## Data Availability

The datasets presented in this study can be found in online repositories. The names of the repository/repositories and accession number(s) can be found in the article/Supplementary Material.
